# Retraction Note: pNaKtide Attenuates Steatohepatitis and Atherosclerosis by Blocking Na/K-ATPase/ROS Amplification in C57Bl6 and ApoE Knockout Mice Fed a Western Diet

**DOI:** 10.1038/s41598-022-13458-w

**Published:** 2022-05-30

**Authors:** Komal Sodhi, Krithika Srikanthan, Perrine Goguet-Rubio, Alexandra Nichols, Amrita Mallick, Athar Nawab, Rebecca Martin, Preeya T. Shah, Muhammad Chaudhry, Saroj Sigdel, Mehiar El-Hamdani, Jiang Liu, Zijian Xie, Nader G. Abraham, Joseph I. Shapiro

**Affiliations:** 1 Departments of Medicine, Surgery, Pathology, and Cardiology, Joan C. Edwards School of Medicine, Marshall University, Huntington, USA; 2 Department of Medicine, New York Medical College, Valhalla, NY 10595 USA

Retraction of: *Scientific Reports* 10.1038/s41598-017-00306-5, published online 15 March 2017

The Editors have retracted this Article. After publication, concerns were raised regarding high similarity in images used in the figures presented in this Article. Specifically:

- Ponceau S staining images in Fig. 2a and b
- Ponceau S staining images in Fig. 2 between lanes 1–3 and lanes 4–6
- Western blots presented for Actin in Fig. 3b and Porin in 3c

The Editors therefore no longer have confidence in the presented data.

Nader G. Abraham agrees to this retraction. Komal Sodhi, Rebecca Martin, Preeya T. Shah, Muhammad Chaudhry, Jiang Liu, and Joseph I. Shapiro do not agree to this retraction. Krithika Srikanthan, Perrine Goguet-Rubio, Alexandra Nichols, Amrita Mallick, Athar Nawab, Saroj Sigdel, Mehiar El-Hamdani, and Zijian Xie have not responded to any correspondence from the Editors about this retraction.

